# Assessment of Hazardous Gaming in children and its dissimilarities and overlaps with Internet Gaming Disorder

**DOI:** 10.3389/fpsyt.2023.1226799

**Published:** 2023-10-27

**Authors:** Sonja Kewitz, Katharina Leo, Florian Rehbein, Katajun Lindenberg

**Affiliations:** ^1^Institute of Psychology, Johannes Gutenberg University Mainz, Mainz, Germany; ^2^Institute for Psychology, Goethe University Frankfurt, Frankfurt am Main, Germany; ^3^Department of Social Work, Münster University of Applied Sciences, Münster, Germany

**Keywords:** IGD, diagnostics, psychological assessment, gaming disorder, clinical sample, school sample

## Abstract

**Background and aims:**

Children have been vastly overlooked in Internet Gaming Disorder (IGD) and Hazardous Gaming research so far. The diagnoses are listed in different ICD-11 chapters (addiction vs. problematic health condition) and are thus considered as distinct constructs. However, screening tools for children do not exist yet. We aimed to investigate the psychometric properties of an existing IGD screening tool modified to also assess Hazardous Gaming in children. Further, we aimed to compare the dissimilarity and overlap between (subclinical) IGD and Hazardous Gaming in children.

**Methods:**

The study analyzed data from a mixed school and clinical sample. Data from *N* = 871 children aged between 8 and 12 years of age (*M* = 10.3, *SD* = 0.90) were analyzed. Data were collected via the Video Game Dependency Scale (CSAS) in its parent report version, which was adapted to assess Hazardous Gaming symptoms in addition to the IGD symptoms. Item analyses and reliability and factor analyses were conducted on the Hazardous Gaming version.

**Results:**

The results show that the adapted CSAS version that assesses Hazardous Gaming symptoms in children mostly shows acceptable psychometric properties. Explorative Factor Analysis (EFA) shows a two-factor structure with one factor of higher order. Additionally, results show that 35.2% of all children meeting the threshold for Hazardous Gaming exclusively meet criteria for Hazardous Gaming but not for (subclinical) IGD. Vice versa, 91.3% of children with IGD also meet the criteria for Hazardous Gaming.

**Discussion:**

Hazardous Gaming and (subclinical) IGD are distinct constructs with some overlaps and might have a temporal relation. We recommend adding four items to assess Hazardous Gaming using the CSAS and further evaluate the validity. The assessment of Hazardous Gaming in children is crucial because it might occur earlier than subclinical or full-syndrome IGD.

## 1. Introduction

**Figure 1:**
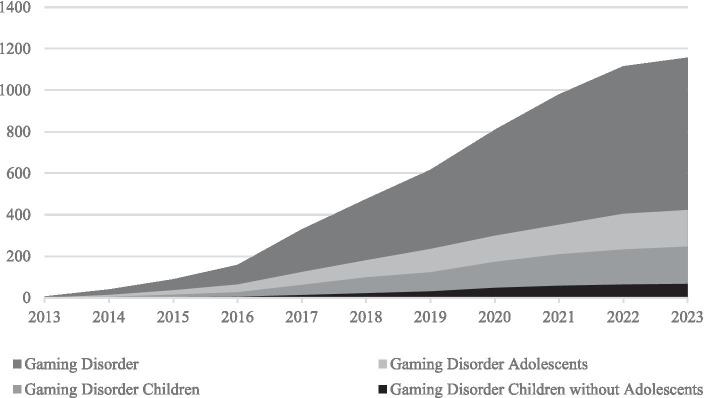
Cumulative published research on Gaming Disorder between 2013 and 2023. Cumulative published papers on PsycInfo between 2013 and 2023 including the terms “‘Gaming Disorder’”, “‘Gaming Disorder’ AND Adolescent*” “‘Gaming Disorder’ AND Child*” or “‘Gaming Disorder’ AND Child* NOT Adolescent*”. The term “Gaming Disorder” was used as it also includes Internet Gaming Disorder. Retrieved May, 9th, 2023 (16).

Internet Gaming Disorder (IGD) has received high scientific interest. IGD is characterized amongst other criteria by loss of control over gaming, prioritizing gaming over other activities, and continuous gaming despite the knowledge of negative consequences (1). Together with gambling disorder, Gaming Disorder is considered a behavioral addiction by the ICD-11 (2). Other proposed (online) behavioral addictions include social-network-use disorder, pornography-use disorder, buying-shopping disorder (3), and streaming disorder (4, 5). The sum of these disorders is often referred to as Internet Addiction. Within these different types of (online) behavioral addictions, overlapping underlying mechanisms have been reported and similar theoretical frameworks have been applied [e.g., (6, 7)]. However, more research is needed to investigate the distinctive features of each behavioral disorder (3). This paper focusses - as stated above - on IGD specifically. In IGD research, children are a population that is often overlooked. In Figure 1 you can see the amount of research on IGD in the database PsycInfo between 2013 and 2023. As depicted, research solely focusing on child age is scarce. Yet, practitioners already observe risky gaming behavior in children. In fact, gaming has become part of many children’s free time at a young age [e.g., (8–10)]. It has been observed that, especially during childhood, gaming time increases with age (8–10). Therefore, childhood seems to be a phase in which people are vulnerable to forming gaming habits. First steps have been taken to analyze diagnostic questionnaires for IGD during childhood (11, 12), which is the basis for validly recording IGD in children. As negative consequences of excessive gaming might not have manifested in children yet (12), it could be even more important to screen children for signs of risks for developing an IGD rather than for criteria of a full-syndrome IGD. To this end one might think of investigating subclinical IGD in children. Subclinical IGD can be understood as the endorsement of some IGD criteria but not sufficient (i.e., five) criteria for a full-syndrome IGD. This means that for subclinical IGD the same criteria as for a full-syndrome IGD apply. The only difference lies within the number of endorsed criteria. Yet, in addition to subclinical IGD, Hazardous Gaming might also be a precursor to IGD.

Therefore, another approach to address this subject would be to consider the construct of Hazardous Gaming [ICD-11 code QE22; (13)] in addition to the assessment of IGD in children. Hazardous Gaming describes a risky gaming pattern instead of a manifested IGD and the diagnosis is characterized by different symptoms to IGD. The threshold for Hazardous Gaming diagnosis is lower than the one for IGD and can be categorized as a “problem associated with health behavior” before a full behavioral addiction has manifested (13). Therefore, it can draw attention to problematic gaming behavior at an earlier stage than IGD can. Being aware of Hazardous Gaming in children might be especially relevant since children are beginning to spend increasingly more time gaming (8–10). If existent in childhood, we would expect that IGD in children is yet in its early stages and therefore assessing lower threshold Hazardous Gaming seems more beneficial in identifying children in need of prevention or (early) intervention. This might prevent children from developing a full IGD during their adolescence or from interferences between Hazardous Gaming with other mental conditions such as depression, anxiety, or peer relationship problems, which are commonly known to occur with problematic gaming [e.g., (14, 15)]. Consequently, Hazardous Gaming can be understood as a complementary perspective to the diagnosis of IGD.

In the ICD-11 Hazardous Gaming describes “a pattern of gaming, either online or offline, that appreciably increases the risk of harmful physical or mental health consequences to the individual or to others around this individual” (13). The risk can result “from [1a] the frequency of gaming, [1b] from the amount of time spent on these activities, [2] from the neglect of other activities and priorities, [3] from risky behaviors associated with gaming or its context, [4] from the adverse consequences of gaming, or from the combination of these” (13). Additionally, the gaming might continue despite the individual being aware of the heightened risk associated with it (13).

To the knowledge of the authors, there are so far no instruments to assess Hazardous Gaming in children and adolescents. To date, only nine search results were identified within the database PsycInfo (16) to investigate Hazardous Gaming at all. The only questionnaire investigating Hazardous Gaming is the Gaming Disorder and Hazardous Gaming Scale (GDHGS) by Balhara et al. (17). This scale shows good psychometric properties for college students, but the wording does not seem applicable to children. The Video Game Dependency Scale [*Computerspielabhängigkeitsskala*; CSAS; (18)] is a scale that validly assesses IGD in adolescents and adults. This scale is repeatedly used in scientific research [e.g., (19–21)] as well as in clinical practice, especially in Germany, because it provides norm values (stanines) which are essential for clinical diagnostics. Furthermore, the self-report version of the CSAS has been evaluated in one of the biggest non-convenience adolescent samples (22, 23). The wording for the questionnaire seems more applicable for children. Additionally, a parental report version for the CSAS exists (18). Since in children parental report is often used as a valid source of information (24), this is a helpful addition in assessing risky gaming behavior in children.

Therefore, we aimed to investigate (a) whether an established questionnaire, measuring IGD in adolescents and adults, is also suitable to measure Hazardous Gaming in German children and/ or (b) whether there are adjustments needed to this questionnaire in order to capture criteria of Hazardous Gaming. On top of that, we aimed to investigate (c) how many children in our study population show symptoms of Hazardous Gaming. Finally, (d) we analyzed the overlap and dissimilarities between (subclinical) IGD and Hazardous Gaming in children.

## 2. Methods

### 2.1. Participants

Our study examined a mixed school and clinical sample of children aged 8 to 12 years. Initially, *N* = 877 children were assessed, *n* = 6 were excluded from analyses due to missing data, resulting in a final sample of *N* = 871 children. The children were on average 10.3 (*SD* = 0.90) years old. Of the sample, 43.5% (*n* = 379) were female, 51.4% (*n* = 448) were male, and 5.1% (*n* = 44) did not specify their gender. The sample consisted of children attending primary or high school, with an overrepresentation of children attending the highest level of German high school (*Gymnasium*), which accounted for 65.8% (*n* = 557) of the sample. Other school types included primary school (*n* = 121) and medium-level high schools (*Realschule*; *n* = 116). The school subsample was composed of *n* = 703 primary school students and fifth graders from the Rhine-Neckar metropolitan region in Germany. Data were retrieved from 39 classes across seven schools as part of the PROTECTdissonance study (25). Data was collected in all schools or classes that were willing to host the prevention program. Therefore, the sample is a convenience sample. Within each participating class, data from all students were collected and analyzed. The data were collected prior to participation in the prevention program PROTECTdissonance. Additionally, *n* = 168 patients from an outpatient clinic in Heidelberg, Germany were included in the study. These data were collected as part of a complete survey conducted by Kewitz et al. (26) in a clinic that treats a variety of psychological disorders without specialization on IGD. All patients with valid data on the CSAS were included in the study. At the time of data collection, *N* = 235 individuals between 8 and 12 years of age were patients at the clinic. Thus, we retained data from 71.5% of all patients at the outpatient clinic between 8 and 12 years of age. The clinical sample included children primarily diagnosed with depressive episodes (3.6%, *n* = 6), neurotic, stress-related, and somatoform disorders (14.9%, *n* = 25), eating disorder, unspecified (0.6%, *n* = 1), habit and impulse disorder, unspecified (0.6%, *n* = 1), disorders of psychological development (4.8%, *n* = 8), as well as behavioral and emotional disorders with onset usually occurring in childhood and adolescence (75.6%, *n* = 127), including hyperkinetic disorders (37.5%, *n* = 63), conduct disorders (10.7%, *n* = 18), and emotional disorders with onset specific to childhood (12.5%, *n* = 21).

We applied no exclusion criteria, and the participants did not receive any form of recompense.

### 2.2. Measures

*(Subclinical) Internet Gaming Disorder* was assessed through the German version of the CSAS (18) by administering the parent report version (CSAS-PR). The questionnaire was completed by the children’s primary caregivers, who were predominantly their biological parents (97.2%). The CSAS was also completed by the children’s stepparents (*n* = 1), grandparents (*n* = 2), foster parents (*n* = 3), and other individuals (*n* = 18, e.g., siblings or educators). A majority of the caregivers who completed the survey were female (75.7%, 18.4% were male, and 6.0% did not disclose their gender). The questionnaire takes approximately 5 to 10 min to complete and evaluates both online and offline gaming behavior using 18 items, with two items per criterion of the DSM-5 diagnosis for IGD. Respondents rate each item on a 4-point Likert scale from 0 “strongly disagree” to 3 “strongly agree,” with only the highest rating counting as endorsement, and only one item needed to fulfil a corresponding criterion. A tentative diagnosis of IGD can be made if at least five out of nine criteria are met, while the presence of two to four criteria indicates a subclinical IGD. The internal consistency for the CSAS in self-report ranges between *α* = 0.92 and *α* = 0.95 (18). The CSAS also assesses the average *gaming time* per day by requesting the parents to estimate their child’s weekday and weekend gaming times. The average gaming time per day was then calculated by combining the two estimates, using the following formula: (5 * average weekday time + 2 * average weekend time)/7.

To measure *Hazardous Gaming*, selected items from the CSAS were re-analyzed in a different manner. The criterion [1a] “excessive gaming frequency” and [1b] “excessive gaming time” were combined into one criterion. Gaming daily was considered excessive gaming frequency. It was assessed by asking how often the child had played video games within the last year on (1) computers, macs, or tablets, (2) game consoles, (3) portable game consoles, and/or (4) mobile phones or smartphones. If the child had played daily on any of these devices, gaming frequency was considered excessive. The frequency was assessed by a 7-point Likert scale ranging from 0 “never” to 6 “daily.” Leaning on the study by Rehbein et al. (27), any amount of average gaming time per day lying in the 90th percentile or above was considered to be excessive. In the current study, 115 min gaming time per day was considered excessive. Thus, the criterion [1] excessive gaming frequency and excessive gaming time was endorsed if a child played on a daily basis at least 115 min per day. The criterion [2] “neglect of other activities and priorities” was endorsed if the IGD criterion “give up other activities” (Item 11 and Item 15) was met. As in the regular analysis of the CSAS, the highest category (“strongly agree”) had to be met on at least one of the two items assessing “give up other activities” for the criterion to be endorsed. The criterion [4] “adverse consequences of gaming” was assessed via the IGD criteria “continue despite problems” (Item 6 and Item 14) and “risk/lose” (Item 16 and Item 18). If at least one of these two criteria was met (i.e., one out of four items), the criterion “adverse consequences of gaming” was considered endorsed. The criterion [3] “risky behaviors associated with gaming or its context” could not be assessed through the CSAS. A recommendation for its future assessment is included in the discussion.

*Sociodemographic variables* that were assessed include age, gender, and school type. Primary diagnoses were solely collected in the clinical sample.

### 2.3. Procedure

The data collection took place via paper-pencil questionnaires that were assigned a pseudonym code. For the school sample, the parents completed the CSAS at home before their children participated in the prevention program. The children brought the questionnaires to school and handed them over to study psychologists. Data for this non-clinical sample were collected from March 2017 to December 2019. For the clinical sample, parents completed the CSAS between April 2018 and November 2019, either at home or on-site at the outpatient clinic. The completed questionnaires were handed over to the responsible psychotherapists, coded with a pseudonym, and passed on to the study coordinator.

### 2.4. Statistical analysis

Psychometric properties of the modified CSAS assessing the Hazardous Gaming criteria “excessive gaming frequency and excessive gaming time,” “neglect of other activities and priorities,” and “adverse consequences of gaming” were analyzed using the mixed school and clinical sample. Descriptive data were analyzed, considering item mean, item standard deviation, item difficulty (*p_i_* = item mean/maximum of scale), and item discrimination. On top of that, the homogeneity and internal consistency were considered. Due to different scaling of the items, standardized Cronbach’s alpha is reported. The analyses were conducted in SPSS, version 29.0.0.0.

Afterwards, an explorative factor analysis (EFA) was conducted using Maximum Likelihood with oblimin rotation. First, the premises for an EFA were analyzed via the Bartlett-Test, the Kaiser-Meyer-Olkin (KMO) coefficient and Measure-of-Sample-Adequacy (MSA) coefficients for each item. A variety of criteria for factor extraction were considered: Parallel analysis, Empirical Kaiser Criterion (EKC), Comparison Data (CD), Maximum-Likelihood (ML) testing, scree plot, BIC, and RMSEA. For interpretation of the factor loadings, confidence intervals were computed to estimate the significance of factor loadings. Additionally, it was tested if a factor of higher order could be retrieved. The EFA was conducted in R, version 4.3.0. The packages psych (28), GPArotation (29, 30), and EFAtools (31) were applied.

Finally, the overlap and dissimilarities between Hazardous Gaming, subclinical IGD, and IGD were examined. Therefore, the percentage of children meeting the threshold for Hazardous Gaming, subclinical IGD, and IGD were considered. Shares of valid data were computed, excluding missing data. For each percentage a 95% confidence interval is given. Finally, Cohen’s Kappa was applied to estimate the overlap in assessment through the different versions of the CSAS. Missing data were not replaced.

### 2.5. Ethics

The Ethics Committee of the Heidelberg University of Education granted approval for the PROTECTdissonance study (EV2019/01). The clinical sample data were assessed as part of a routine care setting, with permission from Heidelberg University’s ethical guidelines. Informed written consent from legal guardians, mainly parents, was obtained, and the study adhered to the principles outlined in the Declaration of Helsinki.

## 3. Results

### 3.1. Item analyses of the modified CSAS assessing Hazardous Gaming

To analyze Hazardous Gaming in children, item analyses of the modified CSAS version were considered first.

#### 3.1.1. Excessive gaming time and frequency

Children played on average *M* = 52 (*SD* = 52, *Min* = 0, *Max* = 463) minutes per day. The most frequent used gaming devices were mobile phones/ smartphones which were being used by 49.2% (*n* = 423) of the children at least multiple times a week, followed by computers which were being used by 32.9% (*n* = 277) of the children at least multiple times a week. The usage frequency per device is given in Table 1.

**Table 1:** Frequency of gaming per device as assessed through the CSAS-PR (23).

Frequency	Computer (*N*)	Game console (*N*)	Portable game console (*N*)	Mobile phone (*N*)
Never	21.6% (182)	40.0% (337)	64.8% (541)	16.2% (139)
1 or 2 times	9.4% (79)	11.9% (100)	9.1% (76)	5.9% (51)
3 to 12 times	12.5% (105)	16.7% (141)	9.7% (81)	12.2% (105)
Multiple times per month	13.7% (115)	11.0% (93)	6.7% (56)	9.5% (82)
Once a week	10.0% (84)	7.5% (63)	3.2% (27)	7.0% (60)
Multiple times a week	26.7% (225)	10.9% (92)	5.3% (44)	33.4% (287)
daily	6.2% (52)	2.0% (17)	1.2% (10)	15.8% (136)

Frequency of gaming was assessed for the past year.

Excessive gaming time was considered endorsed when a child met at least the 90th percentile of gaming time. This equivalated to 115 min per day (*n* = 82). Excessive gaming frequency was considered as daily usage of any gaming device. This criterion was met by 20.5% (*n* = 168) of the children. Daily gaming on any device of at least 115 min per day (i.e., excessive gaming time and excessive gaming frequency) was shown by 6.3% (*n* = 53) of all children.

#### 3.1.2. Neglect of other activities and priorities

Item responses were right skewed and base rates of individuals meeting item cut-offs were low. For item 11 “Because of his/her gaming, my child enjoys other activities less than he/she used to do,” item difficulty was *p_i_* = 0.13 and for item 15 “My child gave up other hobbies or cut down on them because gaming is more important to him/her,” item difficulty was *p_i_* = 0.07. Both items were strongly intercorrelated and moderately correlated with gaming time (see Tables 2 3).

**Table 2:** Item characteristics.

	*N*	Mean	SD	Difficulty *p_i_*	Discrimination for *z*-standardized items
Computer	842	2.9	2.0	0.48	0.21
Game console	843	1.8	1.8	0.30	0.31
Portable game console	835	1.0	1.6	0.17	0.24
Mobile phone	860	3.5	2.1	0.58	0.27
Daily gaming time (in minutes)	764	52	52	0.45^1^	0.52
Item 11	839	0.4	0.8	0.13	0.61
Item 15	840	0.2	0.5	0.07	0.63
Item 6	838	0.2	0.5	0.07	0.62
Item 14	839	0.5	0.8	0.17	0.65
Item 16	837	0.1	0.4	0.03	0.60
Item 18	838	0.1	0.3	0.03	0.44

Computer: frequency of gaming on the computer. Game console: frequency of gaming on game consoles. Portable game console: frequency of gaming on portable game consoles. Mobile phone: frequency of gaming on mobile phones.

^1^Computed with 115 min as maximum (since that is the threshold for excessive gaming time).

**Table 3:** Inter-item correlations.

	Com-puter	Game console	Por-table console	Mobile phone	Gaming time	Item 11	Item 15	Item 6	Item 14	Item 16	Item 18
Computer	–	0.20***	0.10**	0.12***	0.24***	0.20***	0.14***	0.15***	0.18***	0.11**	0.12***
*N*	842	828	819	836	743	812	813	811	812	810	811
Game console	–	–	0.26***	0.25***	0.29***	0.20***	0.15***	0.17***	0.28***	0.16***	0.13***
*N*		843	822	834	746	813	814	812	813	811	812
Portable game console	–	–	–	0.08*	0.17***	0.14***	0.15***	0.16***	0.22***	0.11**	0.09**
*N*			835	830	741	804	805	803	804	802	804
Mobile phone	–	–	–	–	0.39***	0.21***	0.16***	0.19***	0.27***	0.15***	0.11**
*N*				860	758	828	829	827	828	826	827
Gaming time	–	–	–	–	-	0.34***	0.38***	0.33***	0.45***	0.28***	0.13***
*N*					764	740	740	740	739	737	738
Item 11	–	–	–	–	–	–	0.62***	0.42***	0.57***	0.43***	0.40***
*N*						839	839	838	838	836	837
Item 15	-	–	–	–	–	–	–	0.49***	0.54***	0.52***	0.43***
*N*							840	838	839	837	838
Item 6	–	–	–	–	–	–	–	–	0.53***	0.65***	0.36***
*N*								838	837	835	836
Item 14	–	–	–	–	–	–	–	–	–	0.45***	0.28***
*N*									839	837	837
Item 16	–	–	–	–	–	–	–	–	–	–	0.48***
*N*										837	835
Item 18	–	–	–	–	–	–	–	–	–	–	–
*N*											838

*p ≤ 0.05, **p ≤ 0.01, ***p ≤ 0.001. Computer: frequency of gaming on the computer. Game console: frequency of gaming on game consoles. Portable game console: frequency of gaming on portable game consoles. Mobile phone: frequency of gaming on mobile phones.

#### 3.1.3. Adverse consequences of gaming

Least difficult was item 14, “My child often gets into serious fights or arguments at home because he/she spends so much time playing games” (*p_i_* = 0.17), followed by item 6, “Due to his/her frequent gaming, my child sometimes gets in trouble at school or work” (*p_i_* = 0.07), item 16, “My child has already lost or risked an important relationship or friendship because of gaming” (*p_i_* = 0.03), and item 18, “Due to gaming, my child has risked his/her opportunities at school or work” (*p_i_* = 0.03). Item 14 was also most strongly correlated with gaming time (see Table 3). All other descriptive item characteristics can be found in Table 2. For all items, the entire scale was exhausted (for items on usage frequency: *Min* = 0, *Max* = 6, for all other items (excluding gaming time): *Min* = 0, *Max* = 3).

### 3.2. Homogeneity and internal consistency of the modified CSAS assessing Hazardous Gaming

Standardized Cronbach’s alpha was considered to analyze the scale’s reliability. We found evidence for a high reliability (*α* = 0.81, *n* = 693). The mean inter-item correlation laid at *r_ij_* = 0.28 with inter-item correlations ranging from *r* = 0.08 to *r* = 0.65. All inter-item correlations can be found in Table 3.

### 3.3. Factor analyses of the modified CSAS assessing Hazardous Gaming

**Figure 2:**
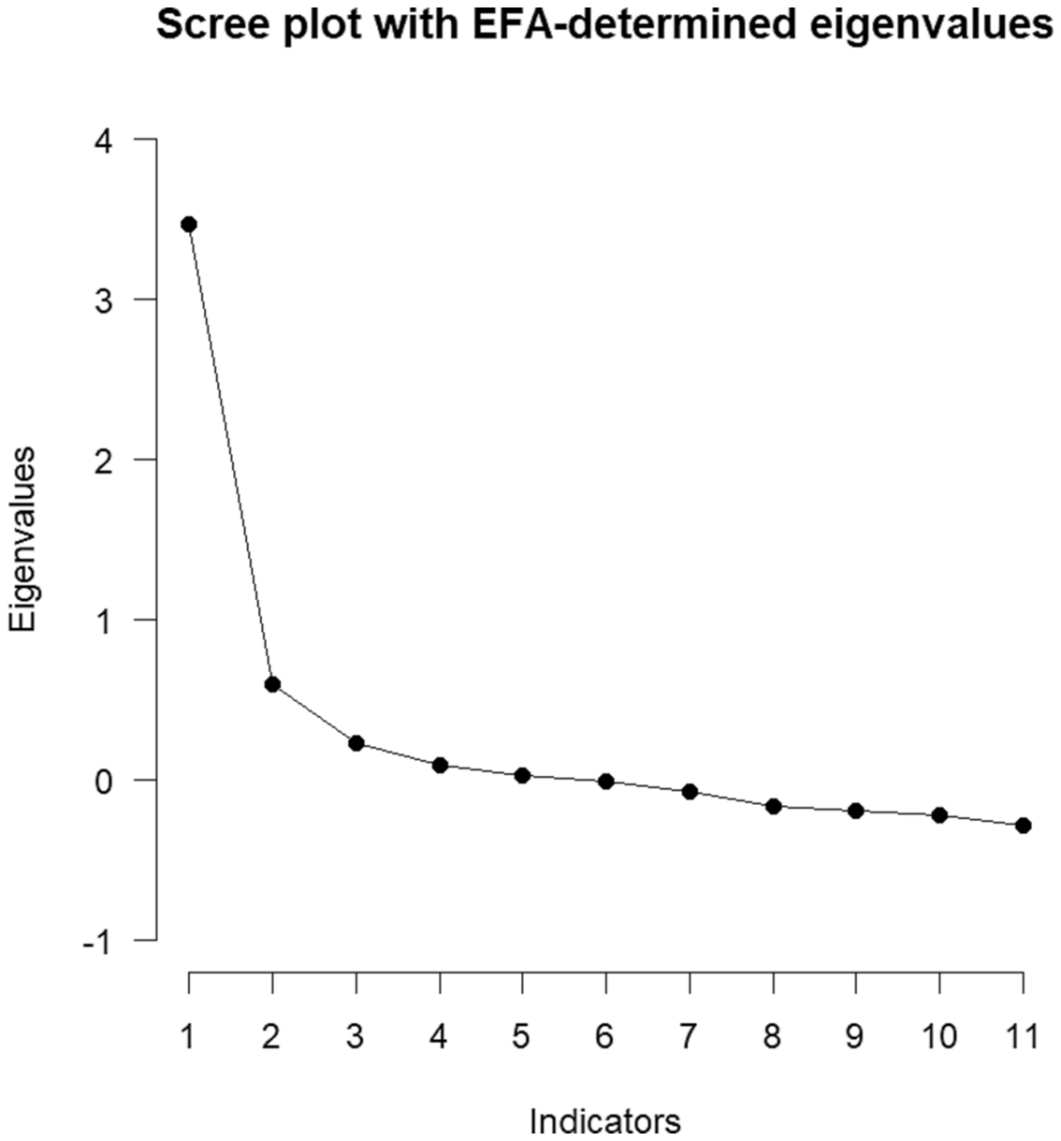
Scree plot of the EFA.

**Figure 3:**
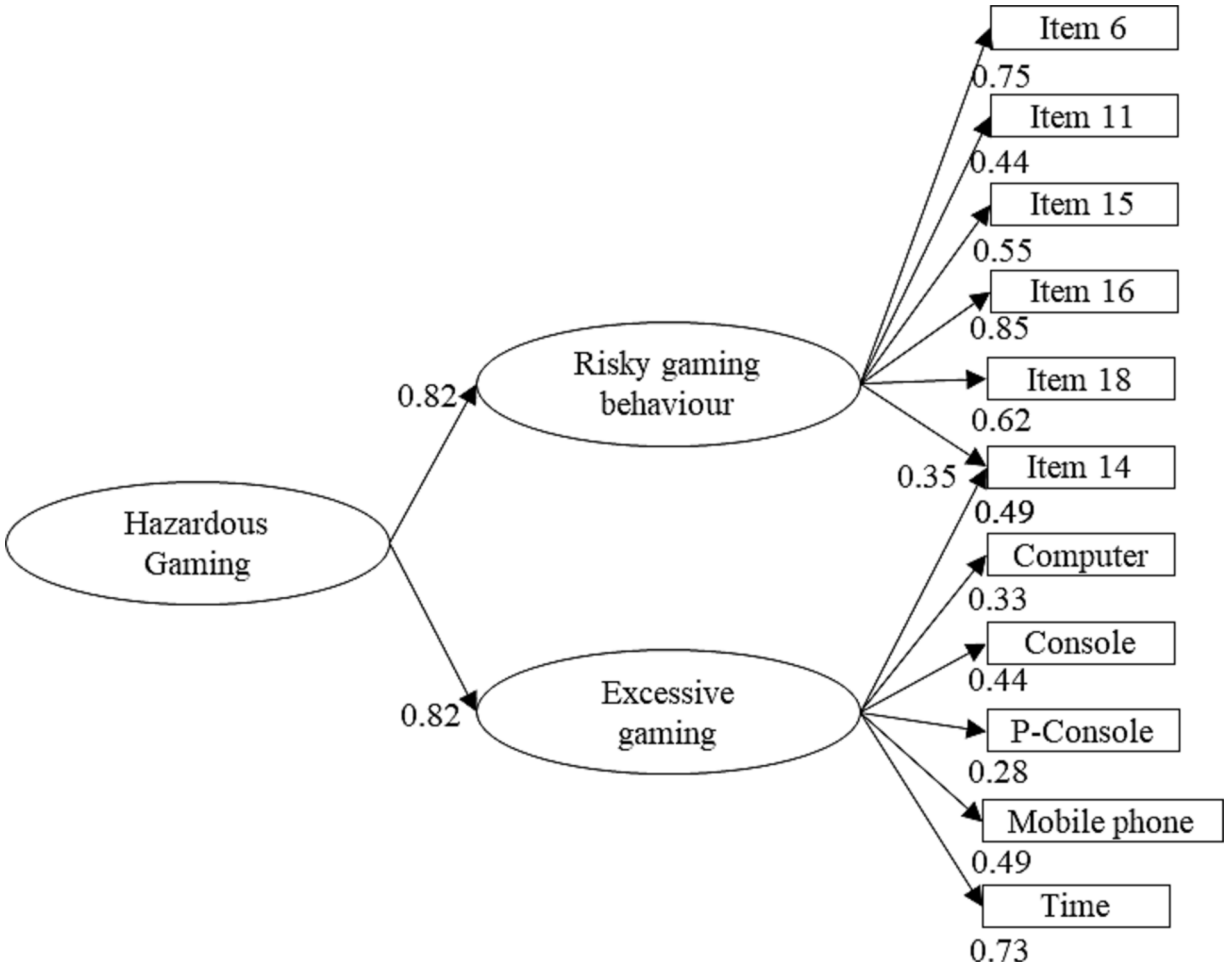
Significant factor loadings. Computer: frequency of gaming on the computer. Game console: frequency of gaming on game consoles. Portable game console: frequency of gaming on portable game consoles. Mobile phone: frequency of gaming on mobile phones.

The factor structure of the modified CSAS to assess Hazardous Gaming was analyzed using EFA. Before conducting the EFA, the premises of an EFA were tested. Since the Bartlett-test reached significance (*χ*^2^ = 2239.53, *df* = 55, *p* > 0.001) and KMO coefficient (KMO = 0.85) as well as MSA coefficients (0.79–0.87) were above 0.50, an EFA could be conducted (32). To determine the number of extracted factors, multiple criteria were considered: EKC and CD suggested the extraction of two factors. The BIC reached a minimum at three factors (BIC = −84.4). Parallel analysis and ML-testing suggested the extraction of four factors (at five factors the *χ*^2^ test did not reach significance anymore, *χ*^2^ (10) = 13, *p* = 0.20), whereas the RMSEA reached its minimum at five factors (RMSEA = 0.02). The scree plot can be found in Figure 2. If different extraction methods do not converge, Auerswald and Moshagen (33) suggest that results of parallel analysis, CD, or EKC can be chosen. Due to facility of interpretation and the convergence of CD and EKC, the two-factor solution was chosen. The first factor’s eigenvalue laid at 2.48, explaining 23% of variance. The second factor’s eigenvalue laid at 1.76, explaining 16% of variance. The factors were correlated (*r* = 0.55). Afterwards, an extraction of a higher order factor was conducted. The extraction of one factor of higher order is suggested by parallel analysis. This factor had an eigen-value of 1.35, explaining 67% of variance. The significant factor loadings of the complete EFA are illustrated in Figure 3. The double loading of Item 14 hinders clear interpretation. Yet, it seems that factor 1 represents risky gaming behavior and factor 2 excessive gaming.

### 3.4. Prevalence, dissimilarities, and overlap of Hazardous Gaming and (subclinical) IGD

**Figure 4:**
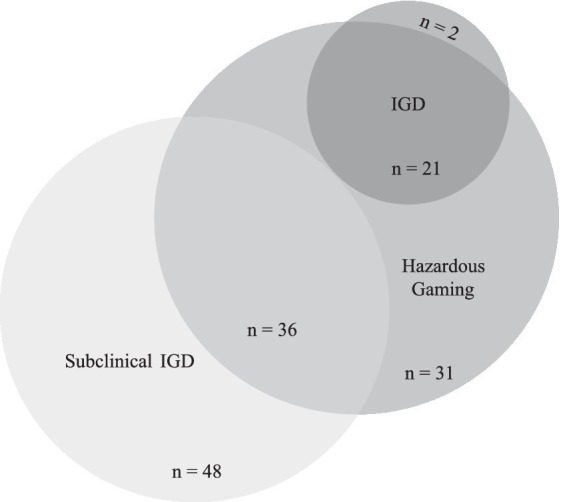
Overlap and dissimilarities between Hazardous Gaming, subclinical IGD, and full-syndrome IGD. The threshold for Hazardous Gaming was met by *n* = 90. However, two of those individuals had too many missing data points to analyze their (subclinical) IGD data. Therefore, only 88 individuals reaching the threshold for Hazardous Gaming are depicted in this figure (excluding the two individuals that have missing data on (subclinical) IGD).

To analyze the overlap between Hazardous Gaming and (subclinical) IGD, we calculated the shares of children fulfilling the threshold for each diagnosis. The threshold for Hazardous Gaming was met by 10.4% (95% CI = 8.3–12.4; *n* = 90) of all children, the threshold for subclinical IGD was met by 10.0% (95% CI = 8.0–12.0; *n* = 84), and the threshold for IGD by 2.7% (95% CI = 1.6–3.8, *n* = 23). Descriptively more boys reached the threshold than girls: Within the male subgroup 15.8% (95% CI = 12.5–19.2, *n* = 71) met the threshold for Hazardous Gaming, 16.3% (95% CI = 12.8–19.8, *n* = 71) for subclinical IGD, and 4.1% (95% CI = 2.3–6.0, *n* = 18) for IGD. In comparison 5.1% (95% CI = 2.8–7.3, *n* = 19) of the girls met the threshold for Hazardous Gaming, 3.3% (95% CI = 1.5–5.1, *n* = 12) for subclinical IGD, and 1.4% (95% CI = 0.2–2.6, *n* = 5) for IGD. The overlap between the different constructs can be seen in Figure 4. In total, 91.3% of the children reaching the threshold for IGD also met the threshold for Hazardous Gaming. Yet only 42.9% of the children that met the threshold for subclinical IGD also met the threshold for Hazardous Gaming. On top of that, 35.2% of all children fulfilling the threshold for Hazardous Gaming neither fulfilled the threshold for IGD nor subclinical IGD, therefore, representing a subgroup of its own.

To quantify the overlap, Cohen’s Kappa was computed to analyze the share of the different constructs. Cohen’s Kappa laid at *κ* = 0.35 (*p* < 0.001) for Hazardous Gaming and subclinical IGD, indicating a fair overlap (34) of the constructs. Also, the similarity between Hazardous Gaming and IGD laid at *κ* = 0.35, (*p* < 0.001). The overlap between any IGD (subclinical or full-syndrome) with Hazardous Gaming laid at *κ* = 0.53 (*p* < 0.001) which corresponds to a moderate similarity (34).

## 4. Discussion

This paper analyzed Hazardous Gaming in children as a relevant health condition which by definition is associated with future psychopathology such as IGD or other mental disorders (13). It is one of the first papers investigating Hazardous Gaming in any age group. To date, only nine search results on “Hazardous Gaming” were identified within the database PsycInfo (16), This approach might be especially promising in a young age group, since based on substance addiction research it is known that earlier onset of harmful use predicts addiction patterns later in life (35). Since prevalence rates of IGD among adolescents are already substantial (36) and children spend a lot of their leisure time gaming (8), it is crucial to address much younger age groups with screenings for Hazardous Gaming. However, instruments to screen for Hazardous Gaming in children do not exist yet. Therefore, this study aimed to test whether Hazardous Gaming in children can be assessed by using specific items from a validated IGD questionnaire (CSAS) that capture Hazardous Gaming symptoms and to assess the psychometric properties of this modified version. Moreover, this study aimed to investigate whether adjustments or additions are needed to capture criteria of Hazardous Gaming using this tool.

Three out of four ICD-11 criteria could be captured with the modified version of the CSAS. The results suggest a good reliability and adequate item difficulty of the adapted version. EFA displayed that a two-factor structure with one factor of higher order showed satisfying results. Thus, the Hazardous Gaming construct seems to be best described by one factor of higher order (Hazardous Gaming) and two factors representing [1] risky gaming behavior (neglect of other activities and adverse consequences of gaming) and [2] excessive gaming (gaming time and frequency). Taken together, it is possible to reliably screen for Hazardous Gaming using an alternative score of the CSAS.

Since our adapted version of the CSAS did not cover all criteria named in the ICD-11, we suggest assessing the criterion “risky behaviors associated with gaming or its context” by including four more items to the CSAS when assessing Hazardous Gaming, displayed in Supplementary Table 1. The suggested items cover the negative impact of gaming on the child’s finances, diet, sleep, and behavior in traffic. The latter focusses on the direct impact of gaming in traffic, e.g., playing a game on a smartphone while walking in the street. This question does not aim to ask for risky behavior in traffic due to Game Transfer Phenomena (37), as this is another complex construct. The set of additional items was generated by the authors on a theoretical basis. Future research should address the assessment of its psychometric properties. To facilitate the calculation of the alternative CSAS score for Hazardous Gaming, we designed a supplementary sheet (see Supplementary Table 2).

Beyond the analysis of the psychometric properties of the modified CSAS version, we aimed to investigate how many children of our study population show symptoms of Hazardous Gaming. Further, we aimed to analyze the overlap and dissimilarities between individuals fulfilling the construct of Hazardous Gaming and (subclinical) IGD.

Although we captured only three out of four Hazardous Gaming criteria and thus might probably underestimate the prevalence (because the criteria are linked by an “or” condition in ICD-11), a relevant share of the child sample (*n* = 90; 10.4%) met the criteria for Hazardous Gaming. Another 10.0% (*n* = 84) met the criteria for subclinical IGD. These populations seem to be largely distinct, represented by a share of 35.2% of children meeting the threshold for Hazardous Gaming exclusively and a share of 57.1% meeting the threshold for subclinical IGD exclusively. Vice versa, 91.3% of children with IGD also met the criteria for Hazardous Gaming.

Hazardous Gaming and subclinical IGD seem to represent two different risk factors for the development of a full-syndrome IGD. Thus, a precursor diagnosis for IGD seems to not only be subclinical IGD but also Hazardous Gaming. One crucial difference between the concepts of Hazardous Gaming and subclinical IGD may be explained by excessive gaming (time and frequency), which is part of the definition of Hazardous Gaming, but not of (subclinical) IGD (1, 13). The results underpin that Hazardous Gaming is not just a sub-construct of IGD but a standalone construct of its own that overlaps with IGD. However, more fundamental research is needed to investigate the validity of the proposed criteria for Hazardous Gaming by the ICD-11.

In clinical practice we suggest screening for children’s risky gaming behavior to avoid full-syndrome IGD (possibly later in life). If risky behavior becomes apparent at an early stage, prevention or early intervention might hinder the onset of a disorder later in life. At this moment, the scientific data cannot give a clear answer whether to prefer screening for subclinical IGD or Hazardous Gaming. It does, however, give insights into some overlaps and differences between the two constructs. Thus, this study paves the way for future studies enabling clear recommendations for clinical practice in the future.

There are some limitations to the adaptability of the CSAS in assessing Hazardous Gaming during childhood: Some items assessing gaming frequency (i.e., computers, portable game consoles, mobile phones) show an item discrimination below 0.30. This is below a desirable threshold (38). The items measuring gaming frequency also show relatively low correlations with other items and some show relatively low factor loadings. On the one hand, the reason for this relatively low suitability might lie within the assessment of gaming frequency through a questionnaire. Daily gaming on any device is the highest resolution for the assessment of gaming frequency possible through the CSAS. Yet, in the current sample 20.5% of the children were gaming daily. Therefore, an even higher resolution for the assessment of gaming frequency might be necessary to assess “excessive” gaming validly. Excessive frequency might also be represented by playing games on multiple devices at the same time. That higher resolution could possibly be assessed by Ecological Momentary Assessment (EMA) multiple times a day. Alternatively, tracking data of electronic devices might be helpful. Though, data from multiple devices would have to be integrated in order to get a valid measure. On the other hand, the lacking psychometric properties of the items measuring gaming frequency might also question the validity of the criterion of excessive frequency. In the current study, gaming frequency is in fact less relevant to assess excessive gaming, compared to gaming time (as can be derived from the factor loadings). Therefore, gaming time might in fact be a sufficient feature to assess excessive gaming. Future research should further investigate the role of excessive gaming frequency in context of Hazardous Gaming.

Additionally, item 14 (“My child often gets into serious fights or arguments at home because he/she spends so much time playing games”) has significant factor loadings on both factors. It is also the least difficult item in assessing risky gaming behavior. It is imaginable that excessive gaming in childhood is often associated with conflicts with parents. These conflicts might arise even if no negative consequences (e.g., trouble in school or sleep problems) have occurred yet. At the same time, conflicts with parents can be regarded as a negative consequence itself and, therefore, this item may be part of both factors.

Finally, the adaptation only used parental report. Even though parental report is often considered crucial in diagnostics for children (24), it does come with some limitations as parents do not monitor their children all day long. Therefore, they might not be able to give valid answers on every item, especially concerning gaming patterns. However, in non-clinical samples, adolescents and their parents gave similar answers on IGD questionnaires (39). At the same time, assessments differed in clinical samples (26, 40). Thus, it would be desirable to test whether the proposed adaptation of the CSAS can also be applied to children in self-report so that both perspectives can be assessed. Additionally, as stated above, tracking or EMA data might be helpful additional ways to validly assess gaming patterns.

## 5. Conclusion

The study was able to show that the CSAS can be applied to children to assess three out of four criteria for Hazardous Gaming and, thus, provide a good screening tool. However, future research should include the suggested fourth criterion to assess “risky behaviors associated with gaming or its context” and further investigate the clinical relevance of the different criteria for the construct of Hazardous Gaming. Hazardous Gaming can be a promising way to make children with problematic gaming behavior visible. This approach might help to avoid stigma of an addiction diagnosis at a young age and give a low threshold alternative.

## Data availability statement

The raw data supporting the conclusions of this article will be made available by the authors, without undue reservation.

## Ethics statement

The studies involving human participants were reviewed and approved by the Ethics Committee of Heidelberg University of Education and by the Ethics Committee of Heidelberg University. Written informed consent to participate in this study was provided by the participants’ legal guardian/next of kin.

## Author contributions

SK was involved in conception and design of the study, collected and analyzed data, and wrote the paper. KLe and FR were involved in conception, design of the study, and writing. KLi conceived and designed the study and was involved in data collection, analysis, writing, and supervision. All authors have read and agreed to the published version of the manuscript.
