# Supplementary material for: Assessment of Hazardous Gaming in children and its dissimilarities and overlaps with Internet Gaming Disorder
Source: Front Psychiatry. 2023 Oct 27;14:1226799. doi: 10.3389/fpsyt.2023.1226799 (PMC10641458; doi:10.3389/fpsyt.2023.1226799)
Supplement: Supplementary file 1 [file Table_1.docx]

**Supplementary Table 1**

Supplementary items to assess “risky behaviours associated with gaming or its context”

| **S. Because of gaming my child has regularly…** | Strongly disagree | Somewhat disagree | Somewhat agree | Strongly agree |
| --- | --- | --- | --- | --- |
| S1) … spent more money than intended? | □ | □ | □ | □ |
| S2) … eaten too much or too little? | □ | □ | □ | □ |
| S3) … slept too little? | □ | □ | □ | □ |
| S4) … shown risky behaviour in traffic (e.g. while riding the bike or walking)? | □ | □ | □ | □ |
