# Supplementary material for: Assessment of Hazardous Gaming in children and its dissimilarities and overlaps with Internet Gaming Disorder
Source: Front Psychiatry. 2023 Oct 27;14:1226799. doi: 10.3389/fpsyt.2023.1226799 (PMC10641458; doi:10.3389/fpsyt.2023.1226799)
Supplement: Supplementary file 2 [file Table_2.docx]

**Supplementary Table 2**

Supplementary sheet to the CSAS for assessing Hazardous Gaming

|  | **Yes** | **Prerequisite for fulfilment of the item** | **Criterion** | **En-dorsed** |
| --- | --- | --- | --- | --- |
| Daily gaming on any device? | □ | Both present? | Excessive gaming frequency and excessive gaming time | □ |
| Daily gaming time: >= 115 minutes? *(5* daily gaming time/ school day) +*  *(2* daily gaming time/ weekend day) / 7* | □ |  |  |  |
| **Answer „3 – strongly agree” to any of the following items?** | | | | |
| Item 11 | □ | At least one endorsed? | Neglect of other activities and priorities | □ |
| Item 15 | □ |  |  |  |
| Item 6 | □ | At least one endorsed? | Adverse consequences | □ |
| Item 14 | □ |  |  |  |
| Item 16 | □ |  |  |  |
| Item 18 | □ |  |  |  |
| Item S1 | □ | At least one endorsed? | Risky behaviours associated with gaming or its context | □ |
| Item S2 | □ |  |  |  |
| Item S3 | □ |  |  |  |
| Item S4 | □ |  |  |  |
| **Number of endorsed criteria for Hazardous Gaming** | | | | **∑** |
| **Tentative diagnostic classification according to ICD-11:**  □ 0 criteria endorsed (Unproblematic Gaming) □ 1-4 criteria endorsed (Hazardous Gaming) | | | |  |
